# Crystal structure of 1,2,3,5-di-*O*-methyl­ene-α-d-xylo­furan­ose

**DOI:** 10.1107/S2056989015020022

**Published:** 2015-10-28

**Authors:** Ioannis Tiritiris, Stefan Tussetschläger, Willi Kantlehner

**Affiliations:** a Fakultät Chemie/Organische Chemie, Hochschule Aalen, Beethovenstrasse 1, D-73430 Aalen, Germany

**Keywords:** crystal structure, acetalation, d-xylose, C—H⋯O hydrogen bonds

## Abstract

The title compound, C_7_H_10_O_5_, was synthesized by reaction of d-xylose with paraformaldehyde. In the crystal, the central part of the mol­ecule consists of a five-membered C_4_O ring with an envelope conformation, with the methine C atom adjacent to the O atom being the flap. The protected O atoms of both cyclic acetal groups are oriented so that the four chiral C atoms of the furan­ose part show an *R* configuration. C—H⋯O hydrogen bonds are present between adjacent mol­ecules, generating a three-dimensional network.

## Related literature   

For the synthesis of 1,2,3,5-di-*O*-methyl­ene-α-d-xylose, see: Schmidt & Nieswandt (1949 ▸). For the synthesis and characterization of chiral 1,3-di­hydro­benzo[*c*]furan derivatives and their inter­mediates, see: Ewing *et al.* (2000 ▸).
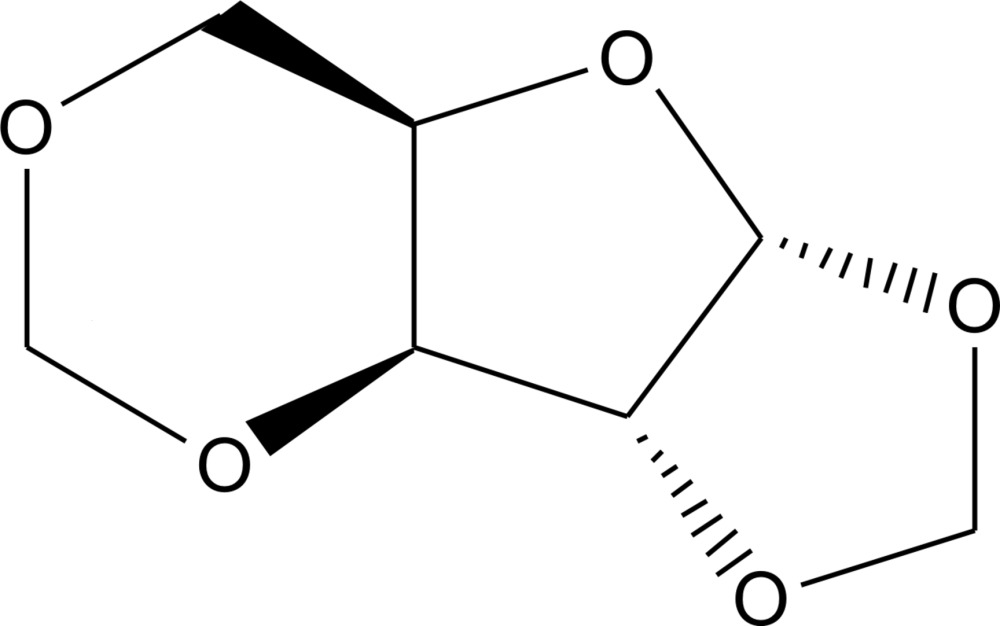


## Experimental   

### Crystal data  

C_7_H_10_O_5_
*M* *_r_* = 174.15
Orthorhombic 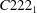

*a* = 8.5509 (11) Å
*b* = 8.6327 (11) Å
*c* = 20.057 (3) Å
*V* = 1480.6 (3) Å^3^
*Z* = 8
Mo *K*α radiation
μ = 0.14 mm^−1^
*T* = 100 K
0.53 × 0.16 × 0.13 mm

### Data collection  

Bruker Kappa APEXII DUO diffractometer
Absorption correction: multi-scan (Blessing, 1995 ▸) *T*_min_ = 0.707, *T*_max_ = 0.744
12973 measured reflections
1858 independent reflections
1667 reflections with *I* > 2σ(*I*)
*R*_int_ = 0.048

### Refinement  

*R*[*F* ^2^ > 2σ(*F* ^2^)] = 0.033
*wR*(*F* ^2^) = 0.074
*S* = 1.05
1858 reflections
110 parameters
H-atom parameters constrained
Δρ_max_ = 0.23 e Å^−3^
Δρ_min_ = −0.20 e Å^−3^

### 

Data collection: *APEX2* (Bruker, 2008 ▸); cell refinement: *SAINT* (Bruker, 2008 ▸); data reduction: *SAINT*; program(s) used to solve structure: *SHELXL97* (Sheldrick, 2008 ▸); program(s) used to refine structure: *SHELXL2014* (Sheldrick, 2015 ▸); molecular graphics: *DIAMOND* (Brandenburg & Putz, 2005 ▸); software used to prepare material for publication: *SHELXL2014*.

## Supplementary Material

Crystal structure: contains datablock(s) I, global. DOI: 10.1107/S2056989015020022/zl2650sup1.cif

Structure factors: contains datablock(s) I. DOI: 10.1107/S2056989015020022/zl2650Isup2.hkl

Supporting information file. DOI: 10.1107/S2056989015020022/zl2650Isup3.cml

**Fig. 1.**
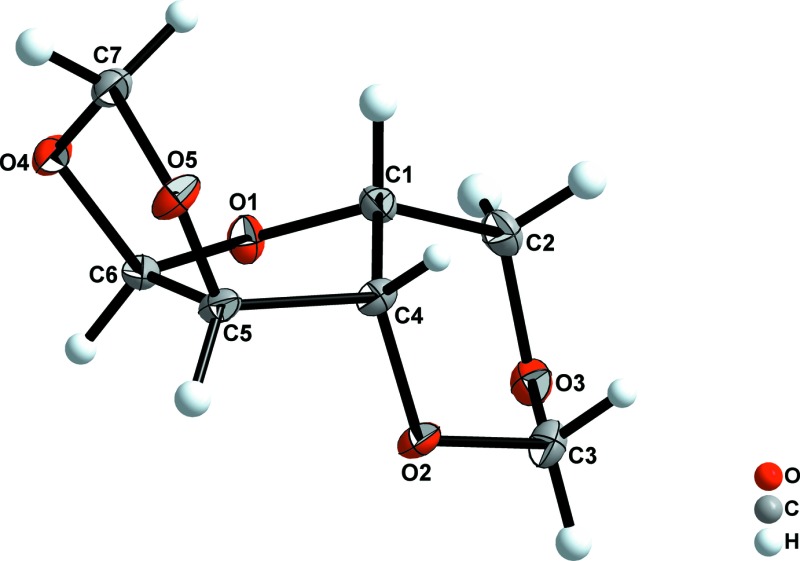
The structure of the title compound with displacement ellipsoids at the 50% probability level.

**Fig. 2.**
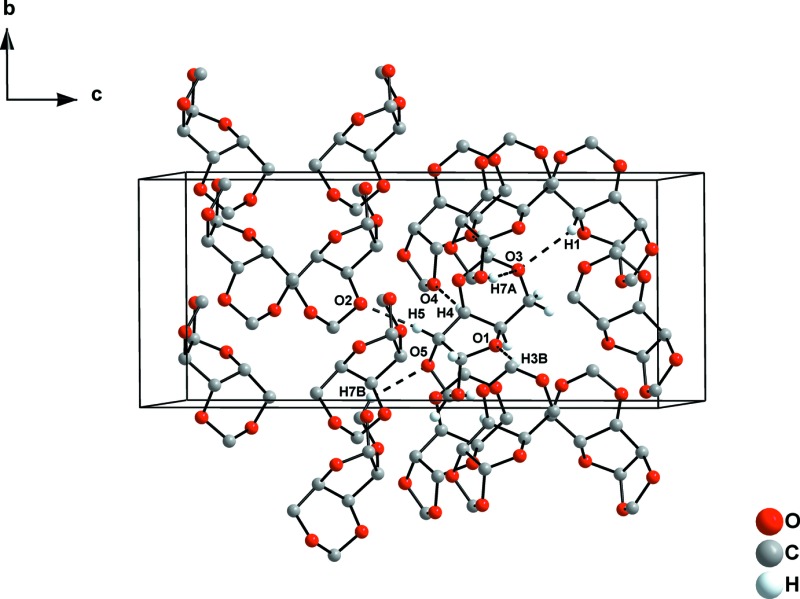
C—H···O hydrogen bonds (black dashed lines) between adjacent molecules in the crystal structure of the title compound (bc view).

. DOI: 10.1107/S2056989015020022/zl2650fig1.tif
The structure of the title compound with displacement ellipsoids at the 50% probability level.

bc . DOI: 10.1107/S2056989015020022/zl2650fig2.tif
C—H⋯O hydrogen bonds (black dashed lines) between adjacent mol­ecules in the crystal structure of the title compound (*bc* view).

CCDC reference: 1432701

Additional supporting information:  crystallographic information; 3D view; checkCIF report

## Figures and Tables

**Table 1:** Hydrogen-bond geometry (, )

*D*H*A*	*D*H	H*A*	*D* *A*	*D*H*A*
C1H1O3^i^	1.00	2.57	3.311(2)	131
C3H3*B*O1^ii^	0.99	2.54	3.458(2)	154
C4H4O4^ii^	1.00	2.46	3.406(2)	157
C5H5O2^iii^	1.00	2.41	3.385(2)	166
C7H7*A*O3^iv^	0.99	2.47	3.337(2)	147
C7H7*B*O5^v^	0.99	2.55	3.390(2)	142

Symmetry codes: (i) 
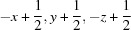
; (ii) 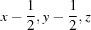; (iii) 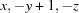; (iv) 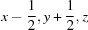; (v) 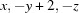.

## supplementary crystallographic information

### S1. Comment

The synthesis of the protected sugar 1,2,3,5-di-*O*-methylene-α-*D*-xylofuranose has been well known for many years (Schmidt & Nieswandt, 1949), its crystal structure, however, remained undetermined. According to the structure analysis, which we would like to now report, the central part of the molecule consists of a five-membered C_4_O ring, which is build by the carbon atoms C1, C4, C5 and C6 and show an envelope conformation (Fig. 1). The protected oxygen atoms of both cyclic acetal groups are oriented in a way so that the four chiral carbon atoms of the furanose part exhibit *R*-configuration. Compounds with similar structures have been obtained as intermediates by using 1,2-*O*-isopropylidene-α-*D*-xylofuranose as a protecting group to synthesize chiral 1,3-dihydrobenzo[*c*]furan derivatives (Ewing *et al.*, 2000). In the crystal structure of the title compound, C—H···O hydrogen bonds between adjacent molecules are present [*d*(H···O) = 2.41–2.57 Å] (Table 1), generating a three-dimensional network (Fig. 2).

### S2. Experimental

According to the literature (Schmidt & Nieswandt, 1949) a mixture of 7.5 g (50 mmol) *D*-xylose and 10.0 g (333 mmol) paraformaldehyde were heated to 373 K. After treating the mixture with 20 g (204 mmol) of concentrated phosphoric acid (85%) and subsequent cooling to room temperature, the mixture has been extracted five times with chloroform. The combined extracts were washed and dried over sodium sulfate. After evaporation of the solvent, the crude product was destilled under reduced presure using a 20 cm *Vigreux* column. The fraction at 363 K (0.1 mbar) contained 3.4 g (39%) of the title compound. Single crystals were obtained by recystallization from petroleum ether and colorless needles were formed suitable for X-ray analysis.

### S3. Refinement

The title compound crystallizes in the non-centrosymmetric space group *C*222_1_; however, in the absence of significant anomalous scattering effects, the Flack parameter is essentially meaningless. The H atoms in CH_2_ and CH groups were placed in calculated positions with d(C—H) = 0.99 Å and d(C—H) = 1.00 Å and refined using a riding model, with *U*(H) set to 1.2 *U*_eq_(C).

### Crystal data

**Table:**

C_7_H_10_O_5_	*D*_x_ = 1.563 Mg m^−^^3^
*M**_r_* = 174.15	Mo *K*α radiation, λ = 0.71073 Å
Orthorhombic, *C*222_1_	Cell parameters from 1667 reflections
*a* = 8.5509 (11) Å	θ = 2.0–28.4°
*b* = 8.6327 (11) Å	µ = 0.14 mm^−^^1^
*c* = 20.057 (3) Å	*T* = 100 K
*V* = 1480.6 (3) Å^3^	Needle, colorless
*Z* = 8	0.53 × 0.16 × 0.13 mm
*F*(000) = 736	

### Data collection

**Table:**

Bruker Kappa APEXII DUO diffractometer	1858 independent reflections
Radiation source: fine-focus sealed tube	1667 reflections with *I* > 2σ(*I*)
Triumph monochromator	*R*_int_ = 0.048
φ scans, and ω scans	θ_max_ = 28.4°, θ_min_ = 2.0°
Absorption correction: multi-scan (Blessing, 1995)	*h* = −11→9
*T*_min_ = 0.707, *T*_max_ = 0.744	*k* = −11→11
12973 measured reflections	*l* = −26→26

### Refinement

**Table:**

Refinement on *F*^2^	Secondary atom site location: difference Fourier map
Least-squares matrix: full	Hydrogen site location: inferred from neighbouring sites
*R*[*F*^2^ > 2σ(*F*^2^)] = 0.033	H-atom parameters constrained
*wR*(*F*^2^) = 0.074	*w* = 1/[σ^2^(*F*_o_^2^) + (0.0335*P*)^2^ + 0.5951*P*] where *P* = (*F*_o_^2^ + 2*F*_c_^2^)/3
*S* = 1.05	(Δ/σ)_max_ < 0.001
1858 reflections	Δρ_max_ = 0.23 e Å^−^^3^
110 parameters	Δρ_min_ = −0.20 e Å^−^^3^
0 restraints	Extinction correction: *SHELXL2014* (Sheldrick, 2015), Fc^*^=kFc[1+0.001xFc^2^λ^3^/sin(2θ)]^-1/4^
Primary atom site location: structure-invariant direct methods	Extinction coefficient: 0.0061 (7)

### Special details

**Table:**

Geometry. All e.s.d.'s (except the e.s.d. in the dihedral angle between two l.s. planes) are estimated using the full covariance matrix. The cell e.s.d.'s are taken into account individually in the estimation of e.s.d.'s in distances, angles and torsion angles; correlations between e.s.d.'s in cell parameters are only used when they are defined by crystal symmetry. An approximate (isotropic) treatment of cell e.s.d.'s is used for estimating e.s.d.'s involving l.s. planes.
Refinement. Refinement of *F*^2^ against ALL reflections. The weighted *R*-factor *wR* and goodness of fit *S* are based on *F*^2^, conventional *R*-factors *R* are based on *F*, with *F* set to zero for negative *F*^2^. The threshold expression of *F*^2^ > σ(*F*^2^) is used only for calculating *R*-factors(gt) *etc*. and is not relevant to the choice of reflections for refinement. *R*-factors based on *F*^2^ are statistically about twice as large as those based on *F*, and *R*- factors based on ALL data will be even larger.

### Fractional atomic coordinates and isotropic or equivalent isotropic displacement parameters (Å^2^)

**Table:**

	*x*	*y*	*z*	*U*_iso_*/*U*_eq_	
O1	0.35776 (16)	0.73794 (16)	0.15438 (7)	0.0209 (3)	
C1	0.2196 (2)	0.6569 (2)	0.17647 (10)	0.0183 (4)	
H1	0.1430	0.7322	0.1957	0.022*	
O2	0.23608 (16)	0.44818 (15)	0.09492 (7)	0.0200 (3)	
C2	0.2642 (3)	0.5386 (3)	0.22854 (11)	0.0250 (5)	
H2A	0.3352	0.5874	0.2614	0.030*	
H2B	0.1689	0.5051	0.2526	0.030*	
O3	0.33921 (17)	0.40599 (17)	0.20058 (7)	0.0224 (3)	
C3	0.2474 (2)	0.3445 (2)	0.14901 (11)	0.0229 (4)	
H3A	0.1413	0.3217	0.1662	0.027*	
H3B	0.2941	0.2459	0.1335	0.027*	
O4	0.28366 (16)	0.95180 (15)	0.08815 (8)	0.0229 (3)	
C4	0.1535 (2)	0.5865 (2)	0.11288 (10)	0.0175 (4)	
H4	0.0381	0.5689	0.1160	0.021*	
O5	0.07534 (18)	0.81726 (16)	0.04937 (8)	0.0239 (4)	
C5	0.1948 (2)	0.7043 (2)	0.05959 (10)	0.0185 (4)	
H5	0.2246	0.6526	0.0168	0.022*	
C6	0.3328 (2)	0.7957 (2)	0.08923 (10)	0.0186 (4)	
H6	0.4287	0.7817	0.0613	0.022*	
C7	0.1181 (2)	0.9509 (2)	0.08583 (11)	0.0213 (4)	
H7A	0.0741	0.9467	0.1314	0.026*	
H7B	0.0788	1.0454	0.0634	0.026*	

### Atomic displacement parameters (Å^2^)

**Table:**

	*U*^11^	*U*^22^	*U*^33^	*U*^12^	*U*^13^	*U*^23^
O1	0.0202 (5)	0.0228 (5)	0.0195 (5)	−0.0066 (4)	−0.0026 (4)	0.0012 (4)
C1	0.0181 (7)	0.0197 (7)	0.0172 (7)	−0.0015 (6)	0.0024 (6)	−0.0010 (6)
O2	0.0258 (5)	0.0129 (5)	0.0213 (5)	0.0043 (4)	−0.0029 (4)	−0.0014 (4)
C2	0.0290 (8)	0.0283 (8)	0.0176 (7)	−0.0006 (7)	0.0028 (6)	0.0026 (6)
O3	0.0213 (5)	0.0241 (5)	0.0217 (5)	0.0026 (4)	−0.0023 (4)	0.0048 (5)
C3	0.0236 (8)	0.0181 (7)	0.0268 (8)	−0.0009 (6)	−0.0026 (6)	0.0042 (6)
O4	0.0200 (5)	0.0141 (5)	0.0347 (6)	−0.0002 (4)	0.0032 (5)	−0.0002 (5)
C4	0.0171 (6)	0.0139 (7)	0.0214 (7)	0.0012 (6)	−0.0024 (6)	−0.0014 (6)
O5	0.0275 (6)	0.0145 (5)	0.0297 (6)	0.0034 (4)	−0.0094 (5)	−0.0004 (5)
C5	0.0245 (7)	0.0149 (7)	0.0162 (7)	0.0041 (6)	−0.0023 (6)	−0.0026 (5)
C6	0.0190 (7)	0.0164 (7)	0.0205 (7)	0.0024 (5)	0.0046 (6)	0.0005 (6)
C7	0.0220 (7)	0.0153 (7)	0.0267 (8)	0.0002 (5)	0.0004 (6)	−0.0015 (7)

### Geometric parameters (Å, º)

**Table:**

O1—C6	1.4147 (18)	C3—H3B	0.9900
O1—C1	1.4429 (17)	O4—C6	1.4119 (17)
C1—C2	1.509 (2)	O4—C7	1.4164 (18)
C1—C4	1.522 (2)	C4—C5	1.517 (2)
C1—H1	1.0000	C4—H4	1.0000
O2—C3	1.4099 (19)	O5—C7	1.4139 (18)
O2—C4	1.4333 (16)	O5—C5	1.4269 (17)
C2—O3	1.4272 (19)	C5—C6	1.539 (2)
C2—H2A	0.9900	C5—H5	1.0000
C2—H2B	0.9900	C6—H6	1.0000
O3—C3	1.4029 (18)	C7—H7A	0.9900
C3—H3A	0.9900	C7—H7B	0.9900
			
C6—O1—C1	109.33 (11)	C5—C4—C1	103.67 (11)
O1—C1—C2	109.50 (12)	O2—C4—H4	112.0
O1—C1—C4	103.92 (11)	C5—C4—H4	112.0
C2—C1—C4	113.81 (12)	C1—C4—H4	112.0
O1—C1—H1	109.8	C7—O5—C5	107.35 (11)
C2—C1—H1	109.8	O5—C5—C4	113.12 (13)
C4—C1—H1	109.8	O5—C5—C6	104.72 (10)
C3—O2—C4	111.67 (11)	C4—C5—C6	104.48 (12)
O3—C2—C1	112.60 (12)	O5—C5—H5	111.4
O3—C2—H2A	109.1	C4—C5—H5	111.4
C1—C2—H2A	109.1	C6—C5—H5	111.4
O3—C2—H2B	109.1	O4—C6—O1	113.30 (12)
C1—C2—H2B	109.1	O4—C6—C5	104.77 (11)
H2A—C2—H2B	107.8	O1—C6—C5	106.97 (11)
C3—O3—C2	109.98 (12)	O4—C6—H6	110.5
O3—C3—O2	111.43 (12)	O1—C6—H6	110.5
O3—C3—H3A	109.3	C5—C6—H6	110.5
O2—C3—H3A	109.3	O5—C7—O4	106.26 (12)
O3—C3—H3B	109.3	O5—C7—H7A	110.5
O2—C3—H3B	109.3	O4—C7—H7A	110.5
H3A—C3—H3B	108.0	O5—C7—H7B	110.5
C6—O4—C7	107.02 (11)	O4—C7—H7B	110.5
O2—C4—C5	105.46 (11)	H7A—C7—H7B	108.7
O2—C4—C1	111.11 (12)		
			
C6—O1—C1—C2	154.82 (12)	O2—C4—C5—O5	151.81 (11)
C6—O1—C1—C4	32.88 (14)	C1—C4—C5—O5	−91.33 (14)
O1—C1—C2—O3	−74.81 (15)	O2—C4—C5—C6	−94.88 (12)
C4—C1—C2—O3	40.99 (18)	C1—C4—C5—C6	21.97 (14)
C1—C2—O3—C3	−52.63 (16)	C7—O4—C6—O1	−94.14 (14)
C2—O3—C3—O2	65.51 (15)	C7—O4—C6—C5	22.11 (15)
C4—O2—C3—O3	−65.43 (15)	C1—O1—C6—O4	96.17 (13)
C3—O2—C4—C5	162.32 (12)	C1—O1—C6—C5	−18.77 (14)
C3—O2—C4—C1	50.63 (15)	O5—C5—C6—O4	−4.41 (15)
O1—C1—C4—O2	79.61 (13)	C4—C5—C6—O4	−123.57 (12)
C2—C1—C4—O2	−39.42 (17)	O5—C5—C6—O1	116.12 (12)
O1—C1—C4—C5	−33.22 (14)	C4—C5—C6—O1	−3.04 (14)
C2—C1—C4—C5	−152.25 (13)	C5—O5—C7—O4	29.22 (16)
C7—O5—C5—C4	98.23 (14)	C6—O4—C7—O5	−32.32 (17)
C7—O5—C5—C6	−14.93 (15)		

### Hydrogen-bond geometry (Å, º)

**Table:**

*D*—H···*A*	*D*—H	H···*A*	*D*···*A*	*D*—H···*A*
C1—H1···O3^i^	1.00	2.57	3.311 (2)	131
C3—H3*B*···O1^ii^	0.99	2.54	3.458 (2)	154
C4—H4···O4^ii^	1.00	2.46	3.406 (2)	157
C5—H5···O2^iii^	1.00	2.41	3.385 (2)	166
C7—H7*A*···O3^iv^	0.99	2.47	3.337 (2)	147
C7—H7*B*···O5^v^	0.99	2.55	3.390 (2)	142

Symmetry codes: (i) −*x*+1/2, *y*+1/2, −*z*+1/2; (ii) *x*−1/2, *y*−1/2, *z*; (iii) *x*, −*y*+1, −*z*; (iv) *x*−1/2, *y*+1/2, *z*; (v) *x*, −*y*+2, −*z*.
