# Supplementary figures and images for: Crystal structure of 1,2,3,5-di-O-methyl­ene-α-d-xylo­furan­ose
Source: Acta Crystallogr E Crystallogr Commun. 2015 Oct 28;71(Pt 11):o889. doi: 10.1107/S2056989015020022 (PMC4645044; doi:10.1107/S2056989015020022)

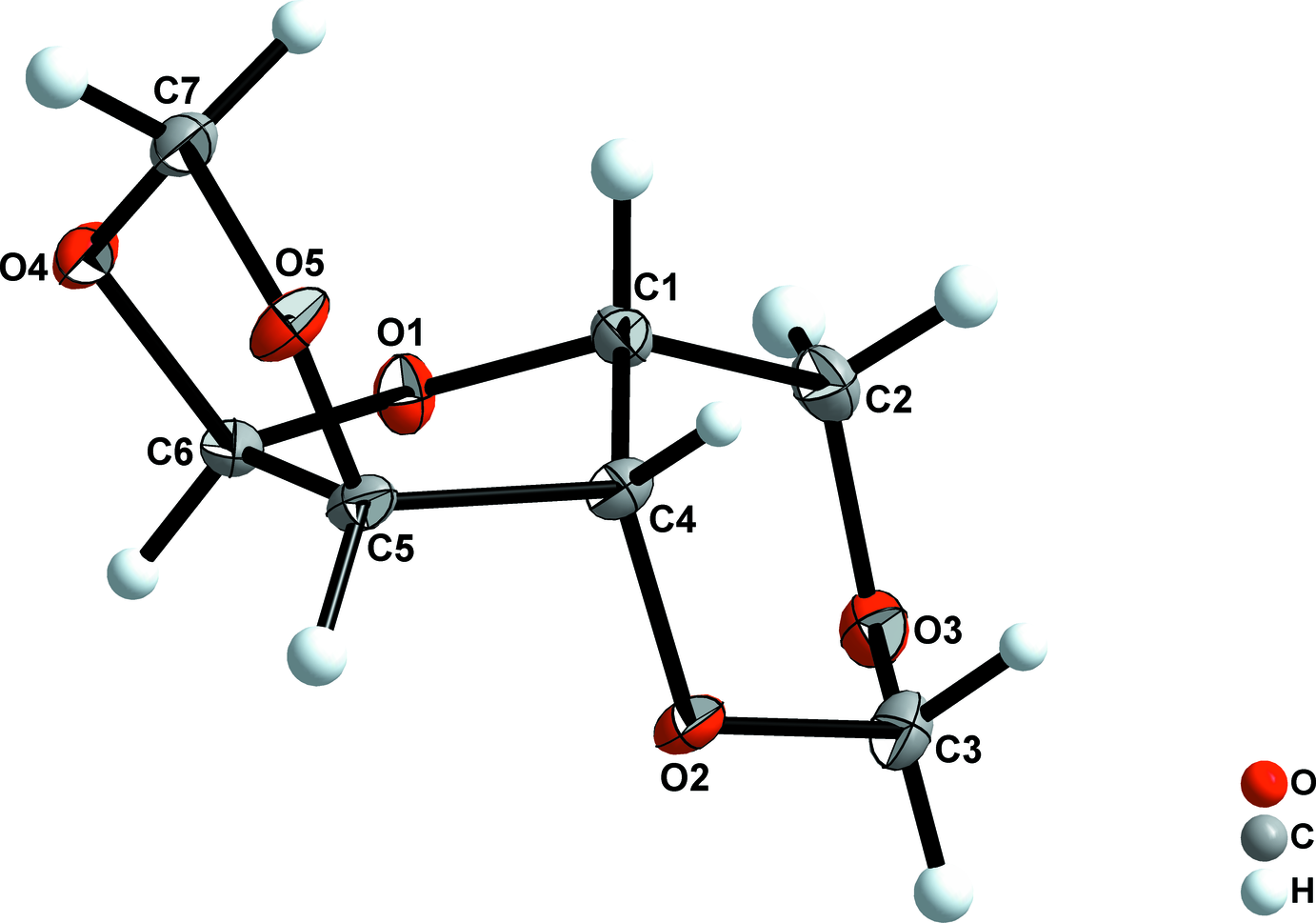

Supplement: Supplementary file 4 [file e-71-0o889-fig1.tif]

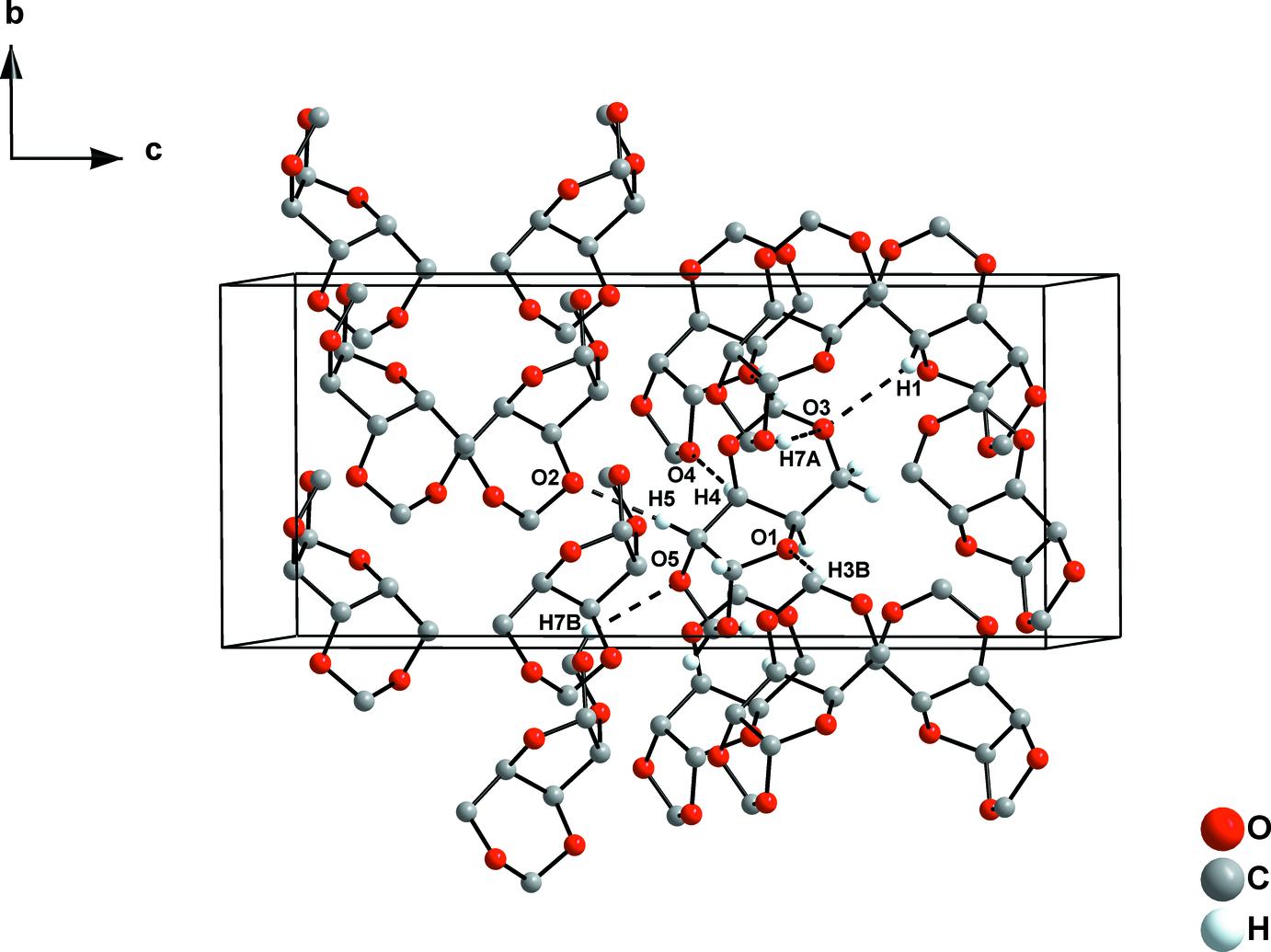

Supplement: Supplementary file 5 [file e-71-0o889-fig2.tif]
